# Efficacy and safety of etravirine-containing regimens in a large cohort of HIV/HCV coinfected patients according to liver fibrosis

**DOI:** 10.7448/IAS.17.4.19574

**Published:** 2014-11-02

**Authors:** Jose Luis Casado, Alvaro Mena, Sara Bañón, Ana Moreno, Angeles Castro, María J Perez-Elías, JD Pedreira, Santiago Moreno

**Affiliations:** 1 Infectious Diseases, Ramon y Cajal Hospital, Madrid, Spain; 2 Internal Medicine, Juan Canalejo, A Coruña, Spain

## Abstract

**Introduction:**

Etravirine has become an alternative in HIV/HCV coinfected patients because of safety and lack of interactions with anti-HCV drugs. The aim of this study was to establish the risk of liver toxicity in HIV/HCV coinfected patients receiving etravirine in the clinical setting, according to the degree of liver fibrosis and different accompanying drugs.

**Material and Methods:**

Cohort study of 211 patients initiating etravirine as part of their antiretroviral regimen. HCV coinfection was defined as a positive RNA-HCV, whereas baseline liver fibrosis was assessed by transient elastography at baseline. Hepatotoxicity was defined as an increased AST/ALT, 5-fold higher over upper, normal limits for patients with normal baseline values, or 3.5-fold if altered at baseline.

**Results:**

HCV coinfection was observed in 145 patients (69%) with a longer time of HIV infection and time on HAART than mono-infected patients, and a lower nadir (182 vs 227 cells/mL; p=0.02) and baseline CD4+ count (446 vs 552 cells/mL; p=0.02). Etravirine was used with two nucleoside analogues in 62%, with boosted darunavir in 17%, with raltegravir in 10%, and with darunavir plus raltegravir or maraviroc in 10% of patients without differences according to HCV serostatus. Transient elastography in 117 patients performed at etravirine initiation (median, 33 days) showed fibrosis 1 and fibrosis 4 in 37% and 24% of cases, and median stiffness value was 8.25 kPa (3.5–69). During an accumulated follow-up of 449.3 patient-years (median, 611 days), only one coinfected patient with fibrosis 4 (stiffness value, 50.1 kPa), receiving a rescue regimen including darunavir/r plus maraviroc plus two nucleoside analogues, developed a grade 3-4 of liver toxicity (0.5%). There were no other episodes of liver toxicity, as defined, and only 6 (3%) and 9 patients (4%) had a grade 1 and 2 of toxicity, respectively, in most cases related to HCV coinfection (6 and 6 cases). Moreover, HCV coinfection or advanced fibrosis was not associated to a higher risk of etravirine discontinuation (26% vs 21%; p=0.27, log-rank test) or virologic failure (9% vs 11%, p=0.56). CD4+ cell count increase was lower in HCV patients (+23 vs +86 at 6 month; p=0.02).

**Conclusions:**

Etravirine is safe in HIV/HCV coinfected patients, even in presence of moderate and advanced liver fibrosis and as part of different antiretroviral regimens.

